# Investigation on Spectrum Estimation Methods for Bimodal Sea State Conditions †

**DOI:** 10.3390/s21092995

**Published:** 2021-04-24

**Authors:** Giovanni Battista Rossi, Francesco Crenna, Marta Berardengo, Vincenzo Piscopo, Antonio Scamardella

**Affiliations:** 1 Department of Mechanical, Energy, Management and Transportation Engineering, University of Genova, Via Opera Pia 15A, 16145 Genova, Italy; francesco.crenna@unige.it (F.C.); marta.berardengo@unige.it (M.B.); 2 Department of Science and Technology, University of Naples “Parthenope”, Centro Direzionale Isola C4, 80143 Naples, Italy; vincenzo.piscopo@uniparthenope.it (V.P.); antonio.scamardella@uniparthenope.it (A.S.)

**Keywords:** sea wave monitoring, bimodal sea state conditions, Welch method, Thomson method, wind sea and swell waves, non-linear least square method

## Abstract

The reliable monitoring of sea state parameters is a key factor for weather forecasting, as well as for ensuring the safety and navigation of ships. In the current analysis, two spectrum estimation techniques, based on the Welch and Thomson methods, were applied to a set of random wave signals generated from a theoretical wave spectrum obtained by combining wind sea and swell components with the same prevailing direction but different combinations of significant wave heights, peak periods, and peak enhancement factors. A wide benchmark study was performed to systematically apply and compare the two spectrum estimation methods. In this respect, different combinations of wind sea spectra, corresponding to four grades of the Douglas Scale, were combined with three swell spectra corresponding to different swell categories. The main aim of the benchmark study was to systematically investigate the effectiveness of the Welch and Thomson methods in terms of spectrum restitution and the assessment of sea state parameters. The spectrum estimation methods were applied to random wave signals with different durations, namely 600 s (short) and 3600 s (long), to investigate how the record length affected the assembled sea state parameters, which, in turn, were assessed by the nonlinear least square method. Finally, based on the main outcomes of the benchmark study, some suggestions are provided to select the most suitable spectrum reconstruction method and increase the effectiveness of the assembled sea state parameters.

## 1. Introduction

The assessment of wave spectra from the analysis of random wave elevations has been a widely investigated topic since the works of Mansard and Funke [1,2] and Battjes and val Vledder [3] because it is a key factor to detect sea state conditions and ensure the safety and navigation of ships [4,5,6]. Really, the assessment of wave spectrum parameters, namely significant wave height, wave peak period, and peak enhancement factor, has been revealed to be a quite-challenging issue since some aspects, such as the selection of a proper spectrum estimation technique, the minimum duration of the wave time signal, and the trade-off between spectral resolution and variance of the spectral estimator represent critical issues of the entire data processing procedure. In fact, spectrum estimation is a key data processing tool for dynamic measurement, and, in the case of sea waves, it also constitutes the first step for estimating sea-state parameters. In view of its importance, a considerable body of literature on the theoretical aspects of spectrum estimation in general [7,8,9,10,11,12,13] and on the practical aspects of its application has been developed in specific investigation areas, including, to some extent, ocean waves [14,15,16]. Two main groups of methods can be identified: non parametrical and parametrical.

The seminal idea under the first group is the “periodogram,” which is the square of the Fourier transform of the signal, divided by the observation duration, originally proposed by Schuster to identify periodicity in noisy signals [17]. The periodogram constitutes a rough estimator of power spectral density (PSD) but provides the basis for more advanced estimators. One of the most effective was formulated by Welch [18] and basically consists of dividing a signal into segments, tapering each segment by a smoothing window, calculating the periodogram of each pre-treated segment, and finally averaging the periodograms. In this way, the quality of the estimate can be highly improved. A noteworthy further development was proposed by Thomson [19] as the so-called multi-taper approach. Further details on these two methods are provided in the following (Section 3), where the proper design of such estimators is also addressed. Parametrical methods (developed since the late 1970s), instead, consist of fitting a general discrete-time model to the series of observations, estimating the parameters of a model and then analytically calculating the corresponding PSD [8]. Two main classes of models have been considered, namely auto-regressive (AR) and auto-regressive-moving-average (ARMA). Basically, the latter typically allows one to reach a good result with lower-order models. On the other hand, though well-established methods—the most popular being Burg’s and the “covariance” methods—exist, different options that are possible for the latter have not well-investigated, and performance may be quite different depending on the application [9,10]. In [20,21], a comparison among parametrical and non-parametrical methods for the estimation of sea-waves spectra was carried out, and the non-parametrical method appeared to be superior. For this reason, in this paper, preceded by a preliminary study presented at an International Congress [22], it was decided to only focus on Welch’s and Thomson’s methods.

Concerning the phenomena to be investigated, based on the review of the actual state of art, it appeared that a variety of efforts have been carried out in the past to detect the sea state parameters of unimodal wave spectra. However, attention still needs to be paid to bimodal wave spectra, which are obtained by the superposition of wind sea and swell components, since issues arise when two wave spectra need to be separately detected based on the spectral analysis of wave elevation time history. Hence, the procedure proposed in [20,21] for unimodal wave spectra was extended to double peak wave spectra, obtained by the superposition of wind sea and swell components with different significant wave heights and peak periods. Particularly, surrogate wave spectra were obtained by starting from a set of reference wave input parameters. They were subsequently used to generate random wave signals with different durations, namely 600 s (short) and 3600 s (long), that were further processed by the two spectrum estimation methods considered in the current analysis to assess the main sea state parameters, namely the significant wave height, the wave period, and the spectrum peak enhancement factor. The assembled sea state parameters were compared with the input values in order to assess which model works better and how the frequency resolution of the two spectral reconstruction methods and the time duration of the wave elevation time history affect the effectiveness of the entire procedure.

The paper is organized as follows. Section 2 provides some basics about bimodal wave spectra and random wave generation. Section 3 focuses on the two spectrum reconstruction techniques used in the current analysis, namely the Welch and Thomson methods, and on the assessment of the sea state parameters by the nonlinear least square method (NLSM), based on the iterative trust-region-reflective algorithm, according to the interior-reflective Newton approach. Section 4 provides the benchmark study, where different combinations of wind sea and swell spectra are selected to investigate the effectiveness of the spectrum reconstruction methods, mainly focusing on the incidence of the record length and the spectrum resolution. A discussion of current results is provided in Section 5, where suggestions in regard to selecting the most suitable spectrum estimation methods, based on the main outcomes of the benchmark study, are made. Finally, conclusions are reported in Section 6, where some suggestions for future works are also provided.

## 2. Input Wave Spectrum and Random Wave Generation

Combined wind sea and swell data are described by a double peak wave spectrum, according to the following equation [23,24]:

(1) $$S\left(\omega \right)=S_{wind}\left(\omega \right)+S_{swell}\left(\omega \right)$$

where ω is the wave circular frequency, while the wind sea and swell components are assumed to be uncorrelated and follow the JONSWAP spectrum $S_{J}\left(\omega \right)$, that in turn, is determined as follows [25]:

(2) $$S_{J}\left(\omega \right)=A_{\gamma }S_{PM}\left(\omega \right)\gamma^{exp\left(-0.5{\left(\frac{\omega -\omega_{p}}{\sigma \omega_{p}}\right)}^{2}\right)}$$

In Equation (2), $\omega_{p}=2\pi /T_{p}$ is the spectral peak frequency depending on the wave peak period $T_{p}$, γ is the peak enhancement factor, and σ denotes the spectral width parameter that is equal to 0.07 if $\omega \leq \omega_{p}$ and 0.09 otherwise. In the same equation, $A_{\gamma }$ is a normalizing factor, depending on the peak enhancement factor:

(3) $$A_{\gamma }=1-0.287ln\left(\gamma \right)$$

where $S_{PM}$ denotes the Pierson–Moskowitz spectrum [26]:

(4) $$S_{PM}\left(\omega \right)=\frac{5}{16}H_{s}^{2}\omega_{p}^{4}\omega^{-5}exp\left(-\frac{5}{4}{\left(\frac{\omega }{\omega_{p}}\right)}^{-4}\right)$$

which, in turn, depends on the significant wave height $H_{s}$ and the wave peak frequency $\omega_{p}$. In absence of additional data, the wave peak period $T_{p}$ can be assessed by the following implicit equation, depending on the wave mean period $T_{m01}$ and the 0th and 1st order spectral moments [27]:

(5a) $$T_{m01}=2\pi \frac{m_{0}}{m_{1}}=2\pi \frac{\int_{0}^{\infty }S_{J}\left(\omega \right)d\omega }{\int_{0}^{\infty }\omega S_{J}\left(\omega \right)d\omega }$$

Equation (5a) can be assembled by the following approximate formulation, which is valid for γ ranging from 1 to 7 and unimodal wave spectra only:

(5b) $$\frac{T_{m01}}{T_{p}}=a+b\gamma +c\gamma^{2}+d\gamma^{3}$$

where a = 0.7303, b  = 0.04936, c = −0.006556, and d = 0.000361 [23]. Equation (5b) was applied to estimate the wave peak periods of the wind sea and swell components of the input bimodal spectra, assumed as reference conditions in the benchmark study carried out in Section 4. In particular, these quantities were assessed starting from the mean wave periods corresponding to the selected Douglas Scale grades and swell categories for the wind sea and swell components, respectively. Hence, in this benchmark study, the wave peak period and the peak enhancement factor of both wind sea and swell components were determined by the best-fit procedure outlined in Section 3.3, starting from the assembled bimodal spectra. The wave mean period was subsequently assessed by Equation (5b), which represents the approximate formulation of Equation (5a). After assessing the combined wind sea and swell spectrum S(ω), the random wave elevation of the bimodal spectrum was determined by Equation (6), based on the superposition of N wave components, each one with circular frequency $\omega_{i}$ and random phase $\varphi_{i}$ [26,27]:

(6) $$\varsigma \left(t\right)=\overset{N}{\underset{i=1}{\sum }}\sqrt{2S\left(\omega_{i}\right)\Delta \omega }cos\left(\omega_{i}t+\varphi_{i}\right)$$

where Δω is the circular frequency interval between two subsequent wave components, satisfying the inequality Δω≤2π/T, to ensure the randomness of the wave signal over time interval T. Equation (6) was used in the current analysis with the main aim of resembling the original bimodal wave spectrum, obtained by the superposition of wind sea and swell components, and going back and forth from the frequency to time domains and vice versa. Particularly, as further detailed in Section 4.1, several random time histories were generated based on different combinations of wind sea and swell spectra and two signal lengths with durations of 3600 s (long) and 600 s (short), respectively.

## 3. Sea Spectrum Estimation

### 3.1. Spectrum Estimation: Welch Method

In this investigation, spectrum estimation was performed both through the Welch and Thomson methods. In the Welch method, also known as the average modified periodogram, the acquired data record of duration T is firstly parsed in segments of duration $T_{0}$ with partial overlap, typically from 20% to 50%. Then, each segment is pre-treated by tapering with a smooth window to reduce the bias due to spectral leakage, and the periodogram (the square of the discrete Fourier transform) is calculated for each of them. The spectrum is obtained by averaging over such periodograms. In this way, bias is reduced by tapering, and variance is limited by averaging. Let us then denote the series of measurements by $x_{i}=x\left(i\Delta t\right)$, where Δt is the sampling interval, i=1,…N, T=NΔt, and $T_{0}=N_{0}\Delta t$. Let $w_{1},\ldots ,w_{N_{0}}$ be a data taper, and then the modified periodogram for the l-th segment is:

(7) $$\;{\hat{S}}_{l}\left(f\right)=\Delta t{\left|\overset{N_{0}}{\underset{i=1}{\sum }}w_{i}x_{i+l-1}e^{-j2\pi fi\Delta t}\right|}^{2}$$

where j is the imaginary unit. The spectral estimator is then:

(8) $$\hat{S}\left(f\right)=\frac{1}{n}\overset{n-1}{\underset{k=0}{\sum }}{\hat{S}}_{km+1}\left(f\right)$$

where n is the number of segments and m is an integer-valued shift factor, satisfying $0<m\leq N_{0}$ and $m\left(n-1\right)=N-N_{0}$ [13].

To apply this method, a proper choice of the analysis features is required [7]. The total observation time, T, is typically fixed by a general experimentation constraint. The remaining features include the kind of taper, the degree of overlap, and the duration of individual segments, $T_{0}$. The goal is to optimise the main “metrological” characteristics of the method, namely its spectral resolution and its variance, or, more clearly, its (relative) standard deviation. The spectral resolution can be understood as the capability to properly represent spectral components, i.e., peaks or, more generally, local maxima, in terms of the localization of the peak maximum and the proper reproduction of its bandwidth. Spectral resolution is (inversely) related to the effective bandwidth of the estimator, in that a large effective bandwidth implies a poor spectral resolution. A small effective bandwidth is therefore desirable. Relative standard deviation instead is a measure of statistical (in)stability.

Regarding the setting of Welch method features, the choice of the degree of overlap is related to the kind of adopted taper, in that the smoother the taper is, the higher the degree of overlap that can be adopted, which results in a larger number of segments with a reduction of the relative standard deviation. On the other hand, the smoother the window is, the larger its bandwidth is and, consequently, the worse its spectral resolution results. Welch suggested adopting a 50% overlap and to apply a cosine (Hanning) window, and this was also the choice made in this study. The effective bandwidth is related both to the taper (*w*, as “window”) and the duration of the individual segments, $T_{0}$. In fact, it can be expressed as $\Delta f_{e}=\alpha_{w}T_{0}^{-1}$, where $\alpha_{w}$ is a factor that depends on the kind of the selected taper and on how bandwidth is defined. In the case of the Hanning window and considering a half-power bandwidth, $\alpha_{w}=1.44$ [11]. Concerning the variance of the estimator with a 50% overlap, a relative standard deviation $u_{S}/S=\sqrt{\left(11/18\right)N_{0}N^{-1}}$ can be assumed, where S denotes the spectrum and $u_{S}$ denotes its absolute standard deviation [18].

These formulae are very useful because they allow one to keep the quality of the result under control. Once the record duration T is fixed and the kind of taper and the degree of overlap have been decided, the duration of the observation window, $T_{0}$, remains the only design parameter to be optimized. Such optimization can be done by a trial-and-error approach, with a trade-off between the need to have a good spectral resolution (which demands a large $T_{0}$) and a small relative uncertainty (which requires a small $T_{0}$) The application of this design criteria to the analysis of sea waves is provided in Section 4.2.

### 3.2. Spectrum Estimation: Thomson Method

This method generalizes the tapering issue by adopting multiple orthogonal tapers, with the aim of recovering information that may be lost when using a single taper. K direct spectral estimators are firstly calculated. Each of them acts on the whole data record by applying a specific taper and then calculating the square of the FFT. The final estimator is the average of such (partial) estimators. In fact, each (partial) estimator is defined by:

(9) $${\hat{S}}_{k}\left(f\right)=\Delta t{\left|\overset{N}{\underset{i=1}{\sum }}h_{i,k}x_{i+l-1}e^{-j2\pi fi\Delta t}\right|}^{2}$$

where $h_{i,k}$ is the kth data taper, usually chosen as the kth discrete prolate spheroidal sequence with parameter W, where 2W is the normalized bandwidth of the tapers, i.e., the bandwidth for Δt=1 s. The final estimator is thus [13]:

(10) $$\hat{S}\left(f\right)=\frac{1}{K}\overset{K-1}{\underset{k=0}{\sum }}{\hat{S}}_{k}\left(f\right)$$

where K is typically chosen to be equal to 2NW−1.

To properly design the analysis, one should consider that the effective bandwidth is now $\Delta f_{e}=2W/\Delta t$ (Hz) and that the estimator is approximately equal in distribution to $S\left(f\right)\chi_{2K}^{2}/2K$, where $\chi_{\nu }^{2}$ is a chi-squared probabilistic variable with ν degrees of freedom. Therefore, a relative standard deviation equal to $K^{-\frac{1}{2}}$ can be assumed [28,29]. In respect to the Welch approach, there is less arbitrariness here because, for a fixed observation time, T, the only parameter to be chosen is the half-bandwidth W, which influences both spectral resolution and relative standard deviation. Again, these design criteria are applied to sea waves in Section 4.2.

### 3.3. Assessment of Sea State Parameters

Sea state parameters, namely the significant wave height $H_{s}$, the wave peak period $T_{p}$, and the peak enhancement factor γ, can be determined by the NLSM, based on the iterative trust-region-reflective algorithm according to the interior-reflective Newton approach [30]. The method, already employed by Rossi et al. [20,21,22], is modified to fit bimodal wave spectra. In this respect, the parameters of bimodal spectra could be accurately detected if the peak frequencies of the wind sea and swell components are far enough to ensure that the two spectral components are sufficiently separated. The assessment of the sea state parameters is based on detecting the minimum of the square residuals [30], according to Equation (11):

(11) $$\bar{x}:\sum_{i}\|S\left(\bar{x},\omega_{i}\right)-S_{i}\|{}^{2}=\underset{x\in X}{\min}\sum_{i}\left(\|S\left(x,\omega_{i}\right)-{\hat{S}}_{i}\|{}^{2}\right)\;$$

where (i) $\bar{x}$ is the vector of the assembled coefficients, namely the significant wave height, the wave peak period, and the peak enhancement factor of wind sea and swell spectra; (ii) x is the tentative vector of sea state parameters, belonging to the vector space $X\subset {ℜ}^{6}$; and (iii) ${\hat{S}}_{i}$ is the estimator of the bimodal wave spectrum at the wave frequency $\omega_{i}$, provided by the estimation methods outlined in Section 3.1 and Section 3.2, on the basis of the random wave history obtained by Equation (6).

## 4. Benchmark Study

### 4.1. Selection of Test Cases

In the numerical study, the wind sea states (reported in Table 1) were considered because they covered a wide range of weather conditions, including both fully (γ=1) and partly (γ>1) developed sea states corresponding to grades 3, 4, 5, and 6 of the Douglas (DG) Scale that, in turn, allowed us to connect the significant wave height with the roughness of sea for navigation. In Table 1, $H_{s}$ denotes the significant wave height and $T_{m01}$ ($T_{p}$) is the mean (peak) wave period. Additionally, two nondimensional parameters are reported. The former is the spectrum peak enhancement factor γ, and the latter is the spectral width parameter ν that is determined according to Equation (12), depending on the 0th, 1st, and 2nd order moments of the wave spectrum:

(12) $$\nu =\sqrt{m_{0}m_{2}/m_{1}^{2}-1}$$

**Figure 1:**
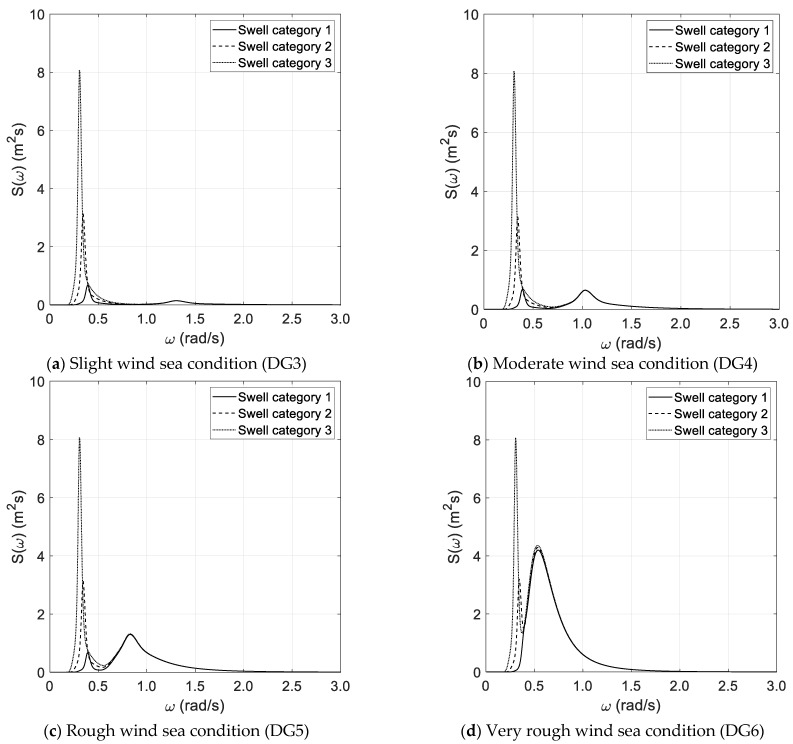
Reference sea state conditions.

Each wind sea state condition is coupled with three swell conditions corresponding to categories 1, 2, and 3, as reported in Table 2. Figure 1a–d reports the reference bimodal spectra investigated in the benchmark study. In this respect, it must be pointed out that the spectral width parameters of wind sea and swell components are each other comparable, as the bimodal spectrum is obtained by the superposition of two JONSWAP wave spectra. 

### 4.2. Spectral Analysis and Sea State Assessment

Spectral analysis was performed by searching optimum values for the analysis parameters, according to the criteria presented in Section 3.2, to propose guidelines for this kind of analysis from the perspective of an online implementation, where nothing is known in advance about the phenomenon under investigation and iterative procedures are not allowed. The optimum choice was different in the two cases of long and of short records. In the case of long duration, Welch analysis was performed with a time-window duration $T_{0}=360\;s$, which corresponded to an effective bandwidth $\Delta f_{e}=0.004\;\mathrm{Hz}$ and a relative standard deviation $u_{S}/S=0.25$. For the Thomson method, instead, a design parameter NW=9, which corresponded to $\Delta f_{e}=0.005\;\mathrm{Hz}$ and $u_{S}/S=0,24$, was chosen. In the case of short duration, two kinds of analyses, with either low (L) or high (H) frequency resolutions, were considered, and the incidence of this selection on the assessment of sea state parameters was investigated. In the low-resolution alternative, for the Welch method, $T_{0}=100\;s$—which corresponded to an effective bandwidth $\Delta f_{e}=0.0144\;\mathrm{Hz}$ and a relative standard deviation $u_{S}/S=0.32$—was chosen. For the Thomson approach, NW=5—corresponding to $\Delta f_{e}=0.0167\;\mathrm{Hz}$ and $u_{S}/S=0.33$—was selected. For the high-resolution case, for the Welch method, $T_{0}=240\;s$—which corresponded to an effective bandwidth $\Delta f_{e}=0.006\;\mathrm{Hz}$ and a relative standard deviation $u_{S}/S=0.49$—was set; for the Thomson approach, NW=2—corresponding to $\Delta f_{e}=0.0067\;\mathrm{Hz}$ and $u_{S}/S=0.58$—was selected.

**Figure 2:**
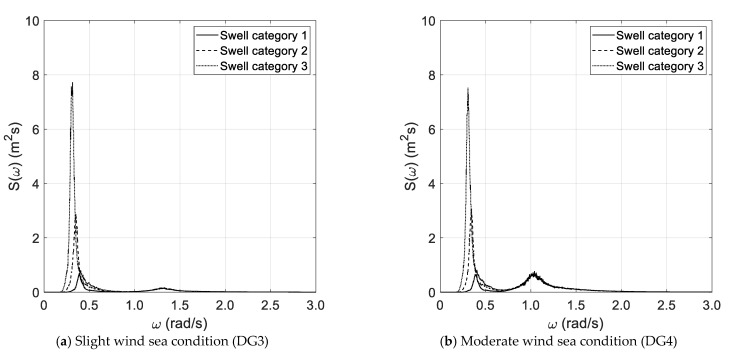

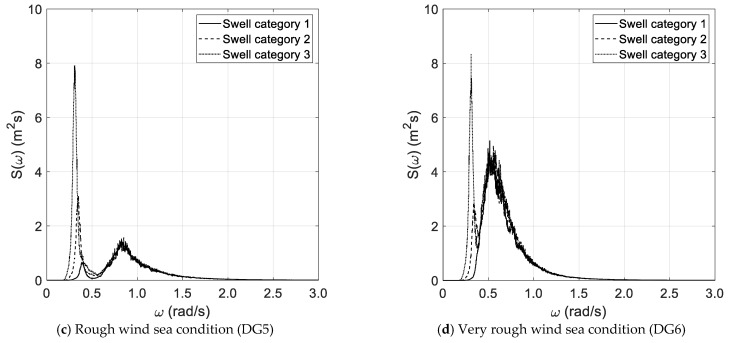
Spectral analysis (Thomson)—long duration.

**Figure 3:**
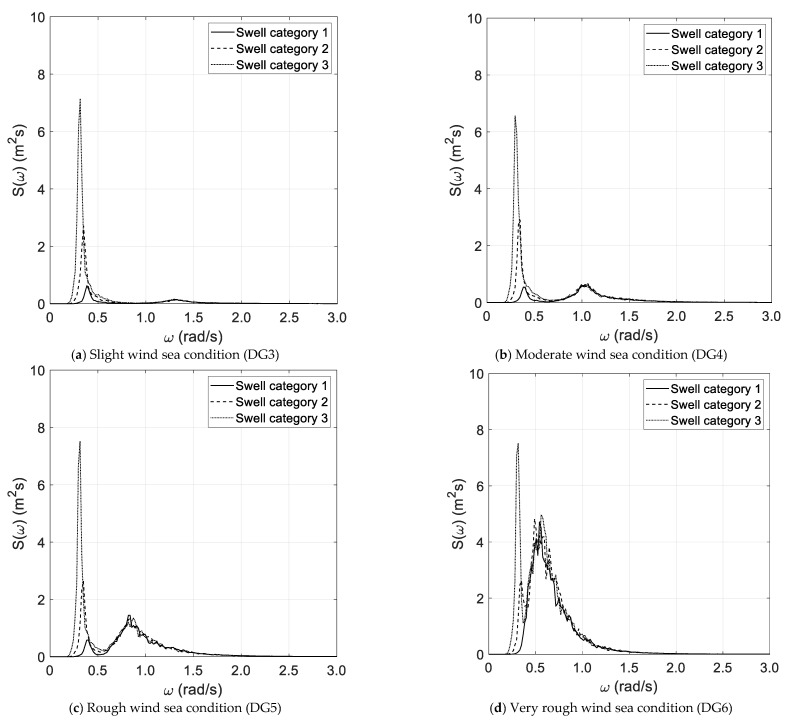
Spectral analysis (Welch)—long duration.

**Figure 4:**
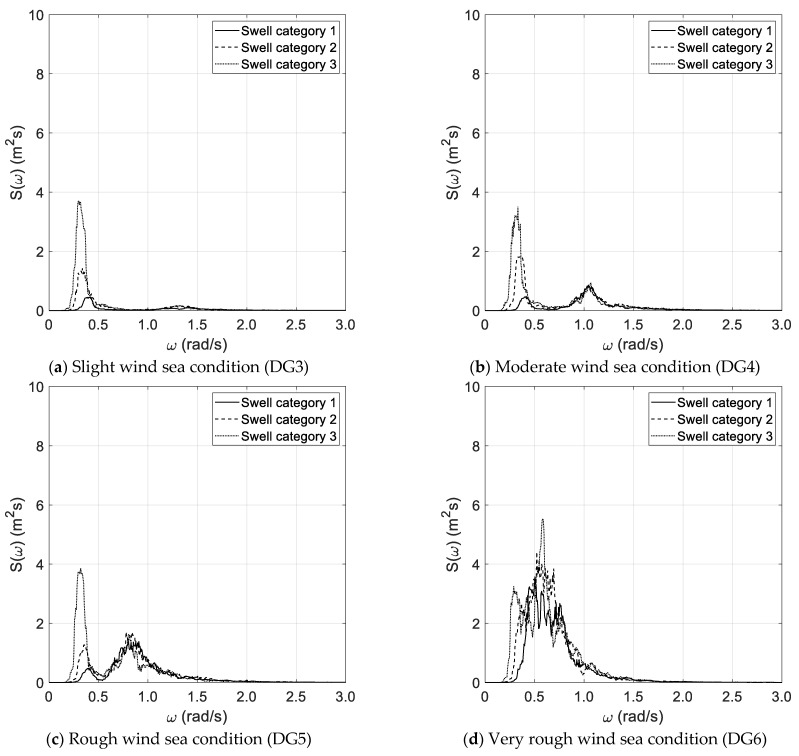
Spectral analysis (Thomson)—short duration with low frequency resolution.

**Figure 5:**
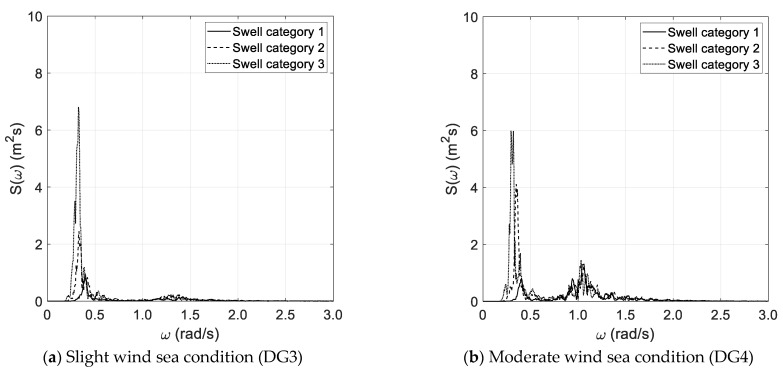

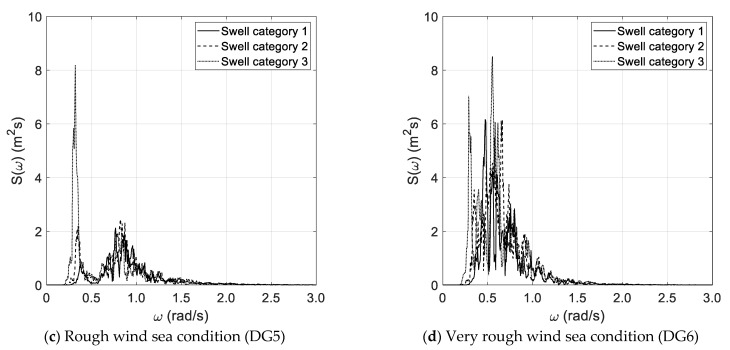
Spectral analysis (Thomson)—short duration with high frequency resolution.

**Figure 6:**
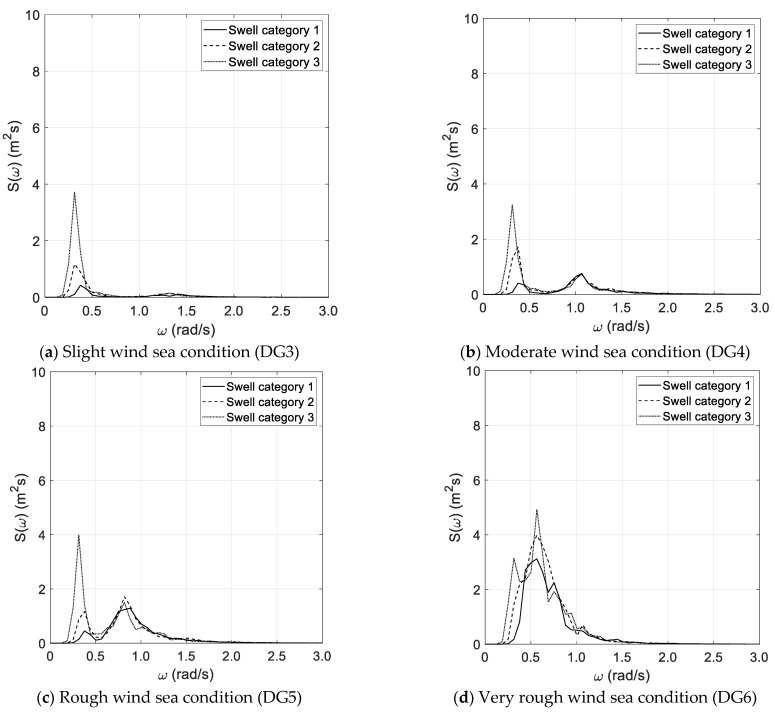
Spectral analysis (Welch)—short duration with low frequency resolution.

**Figure 7:**
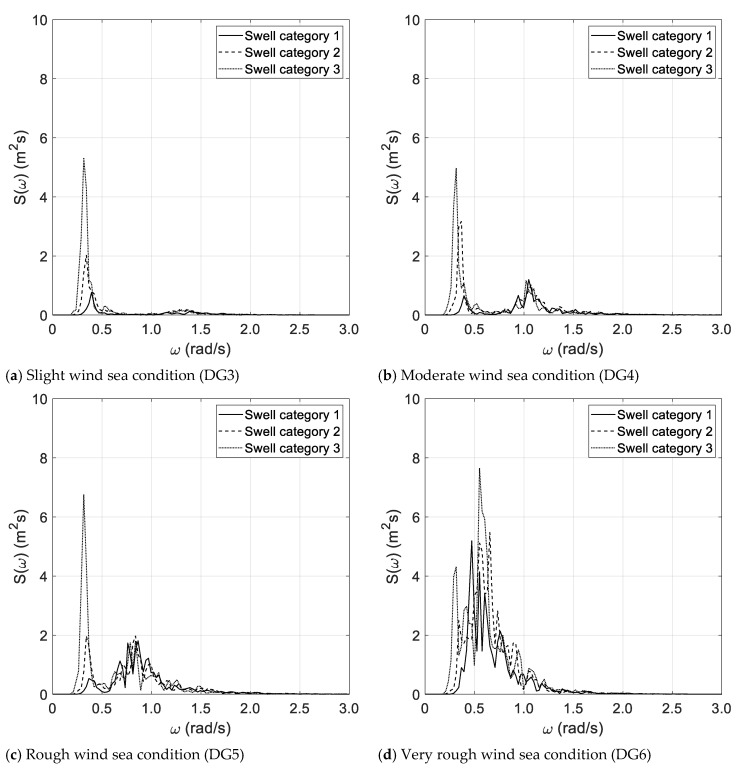
Spectral analysis (Welch)—short duration with high frequency resolution.

The results of the spectral analysis, performed via the Thomson and Welch methods, are outlined in Figure 2a–d and Figure 3a–d, respectively, with reference to the long duration of the random wave history. The results, based on the Thomson method and the short random wave history, are plotted in Figure 4a–d and Figure 5a–d, combined with low and high spectral frequency resolutions, respectively. The same graphs, obtained by the Welch method, are reported in Figure 6 and Figure 7. Each figure refers to a bimodal sea state condition, characterized by a wind sea state corresponding to a DG number ranging from 3 to 6 and coupled with three swell categories ranging from 1 to 3, in compliance with the reference sea state conditions reported in Figure 1a–d.

The assessment of sea state parameters was performed via the NLSM method mentioned in Section 3.3. The significant wave height, the wave peak period, the peak enhancement factor, and the spectral bandwidth of the assembled bimodal spectra, based on the Thomson and Welch methods, are reported in Table 3 and Table 4, respectively, with reference to the long-time duration of 3600 s in terms of percentage variations regarding the initial input parameters. The assembled sea state parameters, based on the 600 s short time recording based on the Thomson methods combined with low and high frequency resolution are listed in Table 5 and Table 6, respectively. Similarly, the sea state parameters, based on the sea spectra obtained by the Welch method are reported in Table 7 and Table 8. It must be pointed out that sea state condition corresponding to DG 6 was not analyzed because the duration of 600 s was not sufficient to achieve a reliable assessment of sea state parameters.

## 5. Discussion

The summary of the benchmark study carried out in Section 4 is outlined in Table 9, which provides the mean absolute percentage variations of the assembled sea state parameters among the various combinations of sea state and swell conditions. In all cases, the average errors were consistent with the errors related to each combination of wind sea and swell conditions. Based on current results, the following main outcomes were achieved:

- Long-time duration: the multi-taper Thomson method was revealed to be slightly superior compared to the Welch one. In fact, the percentage variations of the assembled sea state parameters were lower in all cases, except for the peak enhancement factor of the swell component where almost the results were recognized. In this respect, it must be pointed out that both methods did not predict the peak enhancement factor of the swell component with the same accuracy gained for all the remaining ones, with a mean percentage error equal to about 18%, mainly due to the extremely narrow bandwidth of the swell spectrum component. The same outcomes could be stressed for the spectral bandwidth parameter, as in this case, the Thomson method was also revealed to be slightly superior compared to the Welch one.
- Short-time duration: it must be preliminarily pointed out that the low frequency resolution approach was revealed to be superior in comparison to the high frequency one. In detail, the Welch method was revealed to be slightly superior for the assessment of significant wave height, while the Thomson method provided a better estimation of the wave peak periods and the peak enhancement factors of the both wind sea and swell components. Regarding the spectral bandwidth parameter, no univocal answer was recognized, as the Welch and Thomson methods were revealed to be slightly superior for the wind sea and swell components, respectively.

Based on previous remarks, Table 10 provides the most suitable selection of the spectrum estimation methods depending on the duration of the time history and the frequency resolution of spectral analysis.

The results presented in the table can be used in different ways, depending upon the application and the experimenter’s attitude. One simple approach could be to adopt the method that performs better for the parameters that are considered to be more important. Another more sophisticated approach could be to employ all the methods and relative settings reported in the table and to retain the values provided by the respective best performing set for each wave parameter.

## 6. Conclusions

The paper focused on the application of the Thomson and Welch spectrum estimation methods to assess the main parameters of bimodal wave spectra obtained by the superposition of wind wave and swell components. Two random wave time histories, with 1-h and 10-min durations, were generated based on a set of theoretical bimodal spectra obtained by several combinations of wind sea and swell components that were characterized by different significant wave heights, wave peak periods, and peak enhancement factors. A wide benchmark study was performed to investigate the incidence of the spectrum estimation method on the effectiveness of assembled sea state parameters, obtained by the application of the NLSM to the assembled sea spectra.

Based on the main outcomes of current research, it was gathered that the selection of the spectrum estimation method and the frequency resolution mainly depends on the duration of the time history and the sea state parameter. Particularly, in the case of the 1-h time history, the Thomson method was revealed to be generally superior in comparison to the Welch one. When the duration of the wave time history was short, the low frequency resolution was generally preferable to assess the wind sea and swell parameters when combined with the Welch method for the assessment of the significant wave height and the Thomson method for the evaluation of the wave peak period and the spectrum peak enhancement factor. These outcomes provide guidance for selecting the most suitable spectrum estimation method and frequency resolution in order to improve the assessment of bimodal sea state, also in almost real-time conditions, characterized by an extremely short duration of wave time history. Based on previous remarks, Table 10 provides the most suitable selection of the spectrum estimation methods depending on the duration of the time history and the frequency resolution of spectral analysis. Obviously, these suggestions were based on the main outcomes gathered from the reference sea state conditions that were investigated in the benchmark study discussed in Section 4. Hence, they should not be generalized unless additional analyses and investigation are performed, focusing on different time durations and, eventually, on the endorsement of directional bimodal spectra, with wind sea and swell components characterized by different prevailing directions.

## Figures and Tables

**Table 1:** Selected sea state conditions—wind sea.

DG	Wind Sea Condition	$H_{s}$ [m]	$T_{m01}$ [s]	$T_{p}$ [s]	γ [---]	ν [---]
3	Slight	1.00	4.00	4.82	3.00	0.3285
4	Moderate	2.00	5.00	6.11	2.50	0.3554
5	Rough	3.00	6.00	7.59	1.50	0.3828
6	Very rough	5.00	9.00	11.64	1.00	0.4081

**Table 2:** Selected sea state conditions—swell.

Category	Swell Condition	$H_{s}$ [m]	$T_{m01}$ [s]	$T_{p}$ [s]	γ [---]	ν [---]
1	Small	1.00	14.00	16.12	6.00	0.3515
2	Medium	2.00	16.00	18.32	6.50	0.3482
3	Large	3.00	18.00	20.49	7.00	0.3447

**Table 3:** Sea state assessment (Thomson)—long duration.

DG	$\left|\Delta H_{s,w}\right|$ [%]	$\left|\Delta T_{p,w}\right|$ [%]	$\left|\Delta \gamma_{w}\right|$ [%]	$\left|\Delta \nu_{w}\right|$ [%]	Swell cat.	$\left|\Delta H_{s,s}\right|$ [%]	$\left|\Delta T_{p,s}\right|$ [%]	$\left|\Delta \gamma_{s}\right|$ [%]	$\left|\Delta \nu_{s}\right|$ [%]
3	1.00	0.00	6.00	0.55	1	0.00	0.56	13.17	2.33
	1.00	0.41	2.00	0.06	2	1.00	0.93	21.38	3.96
	0.00	0.41	1.00	0.15	3	2.33	0.00	18.86	3.66
4	0.00	0.00	4.80	0.39	1	0.00	0.37	11.33	1.96
	0.00	0.33	4.80	0.11	2	1.50	0.66	12.00	2.21
	0.50	0.16	2.40	0.14	3	0.33	0.49	25.29	4.99
5	0.67	0.26	1.33	0.10	1	1.00	0.12	13.83	2.39
	0.67	0.79	1.33	0.24	2	0.00	0.16	14.92	2.73
	0.67	0.40	6.67	0.31	3	2.33	0.10	17.71	3.39
6	1.60	2.15	0.00	0.15	1	1.00	0.50	11.33	4.04
	1.00	0.09	0.00	0.05	2	3.00	0.33	34.62	6.81
	0.40	0.52	1.00	0.07	3	1.33	0.10	25.00	4.93

**Table 4:** Sea state assessment (Welch)—long duration.

DG	$\left|\Delta H_{s,w}\right|$ [%]	$\left|\Delta T_{p,w}\right|$ [%]	$\left|\Delta \gamma_{w}\right|$ [%]	$\left|\Delta \nu_{w}\right|$ [%]	Swell cat.	$\left|\Delta H_{s,s}\right|$ [%]	$\left|\Delta T_{p,s}\right|$ [%]	$\left|\Delta \gamma_{s}\right|$ [%]	$\left|\Delta \nu_{s}\right|$ [%]
3	0.00	0.21	5.00	0.37	1	1.00	0.99	12.00	2.11
	1.00	0.21	4.67	0.46	2	2.00	0.93	21.69	4.05
	1.00	0.00	5.67	0.52	3	2.00	0.24	21.57	4.21
4	1.00	0.16	2.80	0.17	1	0.00	0.12	25.50	4.61
	0.50	0.16	4.00	0.42	2	0.50	1.04	5.08	0.95
	2.00	0.00	5.20	0.42	3	0.33	1.07	32.14	6.56
5	1.67	0.53	2.67	0.18	1	3.00	0.37	24.67	4.41
	1.67	0.92	4.67	0.26	2	2.00	0.11	19.38	3.59
	0.67	0.79	7.33	0.26	3	0.00	0.24	1.86	0.35
6	3.00	2.66	0.00	0.20	1	2.00	0.74	16.67	4.27
	1.80	3.09	0.00	0.25	2	1.00	0.66	21.54	6.15
	1.60	2.15	13.00	0.61	3	5.00	0.10	17.14	3.28

**Table 5:** Sea state assessment (Thomson)—short duration and low frequency.

DG	$\left|\Delta H_{s,w}\right|$ [%]	$\left|\Delta T_{p,w}\right|$ [%]	$\left|\Delta \gamma_{w}\right|$ [%]	$\left|\Delta \nu_{w}\right|$ [%]	Swell cat.	$\left|\Delta H_{s,s}\right|$ [%]	$\left|\Delta T_{p,s}\right|$ [%]	$\left|\Delta \gamma_{s}\right|$ [%]	$\left|\Delta \nu_{s}\right|$ [%]
3	4.89	3.87	49.09	3.81	1	0.37	0.01	33.33	6.17
	4.56	0.43	4.19	0.12	2	15.23	3.67	38.46	7.73
	1.64	0.37	0.34	0.12	3	12.35	0.58	42.86	9.02
4	3.78	1.06	26.96	2.50	1	1.58	2.20	33.33	6.06
	6.34	0.99	3.17	3.55	2	1.90	1.32	38.46	7.58
	2.59	1.51	40.33	3.66	3	16.68	1.23	42.86	9.02
5	4.82	1.97	16.71	1.20	1	2.49	0.05	33.33	6.17
	2.44	0.71	28.77	1.23	2	18.55	3.19	38.46	7.52
	8.55	2.81	24.89	0.94	3	9.39	1.60	42.86	8.99

**Table 6:** Sea state assessment (Thomson)—short duration and high frequency.

DG	$\left|\Delta H_{s,w}\right|$ [%]	$\left|\Delta T_{p,w}\right|$ [%]	$\left|\Delta \gamma_{w}\right|$ [%]	$\left|\Delta \nu_{w}\right|$ [%]	Swell cat.	$\left|\Delta H_{s,s}\right|$ [%]	$\left|\Delta T_{p,s}\right|$ [%]	$\left|\Delta \gamma_{s}\right|$ [%]	$\left|\Delta \nu_{s}\right|$ [%]
3	6.13	5.67	40.46	2.19	1	6.19	0.65	16.67	2.65
	5.31	0.27	2.12	1.49	2	8.36	3.50	38.46	7.73
	2.25	0.68	15.83	1.37	3	4.44	3.25	22.01	4.21
4	4.84	1.67	46.69	4.16	1	6.28	1.84	1.25	0.14
	6.21	2.01	22.28	6.70	2	9.69	2.17	7.69	1.35
	4.06	1.45	80.04	6.58	3	12.16	0.98	15.09	2.90
5	5.24	1.77	12.50	0.94	1	9.40	2.42	33.33	6.26
	1.09	0.03	60.06	3.34	2	11.29	0.45	36.42	7.15
	8.92	3.07	63.08	2.90	3	0.31	2.07	19.16	3.66

**Table 7:** Sea state assessment (Welch)—short duration and low frequency.

DG	$\left|\Delta H_{s,w}\right|$ [%]	$\left|\Delta T_{p,w}\right|$ [%]	$\left|\Delta \gamma_{w}\right|$ [%]	$\left|\Delta \nu_{w}\right|$ [%]	Swell cat.	$\left|\Delta H_{s,s}\right|$ [%]	$\left|\Delta T_{p,s}\right|$ [%]	$\left|\Delta \gamma_{s}\right|$ [%]	$\left|\Delta \nu_{s}\right|$ [%]
3	3.39	3.26	52.99	4.44	1	6.26	2.26	18.46	3.16
	3.76	0.04	8.50	0.03	2	6.56	1.42	38.46	7.67
	1.55	0.42	0.55	0.15	3	13.96	2.22	42.86	9.11
4	3.13	0.47	23.09	2.05	1	9.80	3.12	4.42	0.63
	5.27	1.36	3.66	2.90	2	13.24	0.51	7.69	1.32
	2.01	1.69	31.23	2.95	3	19.27	2.22	42.86	9.11
5	5.01	2.35	12.67	1.04	1	11.51	2.14	33.33	6.06
	2.97	0.83	38.49	1.38	2	5.79	0.80	38.46	7.61
	5.57	3.40	27.91	1.02	3	11.31	2.22	42.86	9.11

**Table 8:** Sea state assessment (Welch)—short duration and high frequency.

DG	$\left|\Delta H_{s,w}\right|$ [%]	$\left|\Delta T_{p,w}\right|$ [%]	$\left|\Delta \gamma_{w}\right|$ [%]	$\left|\Delta \nu_{w}\right|$ [%]	Swell cat.	$\left|\Delta H_{s,s}\right|$ [%]	$\left|\Delta T_{p,s}\right|$ [%]	$\left|\Delta \gamma_{s}\right|$ [%]	$\left|\Delta \nu_{s}\right|$ [%]
3	5.37	6.30	35.02	1.37	1.00	3.69	0.33	12.18	1.93
	7.53	0.62	0.81	0.91	2.00	8.07	2.86	38.46	7.73
	3.59	0.22	10.73	1.16	3.00	4.93	3.72	36.96	7.51
4	2.50	2.32	54.33	4.90	1.00	3.35	1.37	13.01	2.22
	8.15	2.13	16.71	8.10	2.00	7.80	2.43	7.69	1.38
	3.35	1.55	23.20	7.96	3.00	12.48	1.05	23.68	4.67
5	5.99	1.95	11.01	0.89	1.00	7.41	2.03	33.33	6.23
	1.63	0.37	38.43	1.36	2.00	7.43	1.73	38.46	7.58
	8.79	3.53	24.10	0.81	3.00	2.77	2.20	32.92	6.64

**Table 9:** Results summary.

Duration	Method	Frequency Resolution	$\left|\Delta H_{s,w}\right|$	$\left|\Delta T_{p,w}\right|$	$\left|\Delta \gamma_{w}\right|$	$\left|\Delta \nu_{w}\right|$	$\left|\Delta H_{s,s}\right|$	$\left|\Delta T_{p,s}\right|$	$\left|\Delta \gamma_{s}\right|$	$\left|\Delta \nu_{s}\right|$
			[%]	[%]	[%]	[%]	[%]	[%]	[%]	[%]
Long	Thomson	High	0.62	0.46	2.61	0.19	1.15	0.36	18.29	3.62
	Welch	High	1.33	0.91	4.58	0.34	1.57	0.55	18.27	3.71
Short	Thomson	Low	4.40	1.52	21.61	1.90	8.73	1.54	38.22	7.59
	Thomson	High	4.90	1.85	38.12	3.30	7.57	1.93	21.12	4.00
	Welch	Low	3.63	1.53	22.12	1.78	10.85	1.88	29.93	5.97
	Welch	High	5.21	2.11	23.82	3.05	6.44	1.97	26.30	5.10

**Table 10:** Selection of spectrum estimation methods (H: high frequency; L: low frequency).

Duration	$H_{s,w}$ [m]	$T_{p,w}$ [s]	$\gamma_{w}$ [---]	$\nu_{w}$ [---]	$H_{s,s}$ [m]	$T_{p,s}$ [s]	$\gamma_{s}$ [---]	$\nu_{s}$ [---]
Long	Thomson							
Short	Welch (L)	Thomson (L)		Welch (L)	Welch (L)	Thomson (L)	Thomson (H)	

## Data Availability

The data presented in this study are available on request from the corresponding author.
